# Impact of Bariatric Surgery on the Healthy Eating Index, Binge Eating Behavior and Food Craving in a Middle Eastern Population: A Lebanese Experience

**DOI:** 10.3390/healthcare9111416

**Published:** 2021-10-21

**Authors:** Jana Jabbour, Dalia Awada, Nour Naim, Ayoub Al-Jawaldeh, Houssein Haidar Ahmad, Hussein Mortada, Maha Hoteit

**Affiliations:** 1 Nutrition Department, School of Health Sciences, Modern University for Business and Sciences, Beirut 6573, Lebanon; jjabbour@mubs.edu.lb; 6 PHENOL Research Group (Public Health Nutrition Program-Lebanon), Faculty of Public Health, Lebanese University, Beirut 6573, Lebanon; 2 Faculty of Public Health, Lebanese University, Beirut 6573, Lebanon; dalia.awada@hotmail.com (D.A.); nour29.nn29@gmail.com (N.N.); 3 World Health Organization Regional Office for the Eastern Mediterranean, Cairo 11371, Egypt; aljawaldeha@who.int; 4 Division of Surgery, Saint George Hospital University Medical Center, Hadath 6573, Lebanon; houshaid@hotmail.com; 5 Faculty of Science, Lebanese University, Zahle 6573, Lebanon

**Keywords:** bariatric surgery, healthy eating index, binge eating behavior, food craving, Lebanon

## Abstract

Even though bariatric surgeries (BS) are on the rise in Lebanon and the Middle East, the changes in diet quality, binge eating, and food cravings in this region are poorly studied peri-operatively. This cross-sectional study aimed to assess binge eating behaviors, food craving and the Healthy Eating Index (HEI) in Lebanese patients who underwent BS in a duration that exceeds 6 months. Evaluation included a dietary assessment of usual diet preoperatively and postoperatively. It included the collection of information on sociodemographic, anthropometric and surgical variables, as well as the administration of dietary recalls and questionnaires to calculate the HEI score, the Binge Eating Scale (BES) and the Food Craving Inventory (FCI). Participants (*n* = 60) were mostly females (85%) who had undergone sleeve gastrectomy (90%), with a mean duration since BS of 2.4 ± 1.8 years. Despite improvements in their HEI scores, 97% of the participants remained in the worst category. The frequency of participants in the severe BES category dropped markedly postoperatively from 78% to 5% (*p* < 0.01). Food craving followed a similar trend, with scores dropping from 50 ± 36 pre-surgery to 30 ± 25 post surgery (*p* < 0.01). Weight regain, prevalent among 40% of participants, was predicted by BES. Despite the improvement in BES and FCI, HEI improvement remained shy. Future interventions should validate findings in other countries and assess means for optimizing HEI scores among BS patients in the Middle East region.

## 1. Introduction

Obesity has been weighing more heavily on individuals, communities, and health care systems over the last decades [1]. Bariatric surgery (BS), recommended when noninvasive lifestyle interventions fail, is the most efficient treatment approach for weight management, and has been associated with a reduction in cardiovascular diseases, cancer incidence and mortality, as well as an improvement in quality of life and self-esteem [2,3,4,5,6]. Despite the imminent short-term benefits of BS, weight regain and disease recurrence remain a reality to many. It has been estimated that only 40% of BS patients maintain 30% of weight loss 12 years after the surgery [7,8,9]. Poor adherence to a Mediterranean diet and having a low Healthy Eating Index (HEI) have been linked to weight regain post operatively [10,11]. Other culprits of weight regain include eating disorders, and limited follow up with the multidisciplinary team [9,11,12,13,14,15,16].

Binge eating disorder (BED) is defined by the loss of control over food consumption that may be accompanied with an intake of large amounts of food. Binge eating affects around 30% of the obese population and 17–48% of BS candidates preoperatively [17,18,19]. Maladaptive food-eating behavior tends to gradually decrease postoperatively and increase thereafter, with a noteworthy emergence of de-novo cases [20]. BED in the BS population has been linked with weight regain, depressive symptomology, the female gender, younger age and lower self-esteem [20,21,22,23,24]. Food craving is the intense desire to consume a food item or beverage that results in compulsive behavior, mimicking addictive conditions [25,26]. Food craving has been associated with eating disorder psychopathology and with obesity [27]. A recent systematic review analyzing food craving among BS patients revealed that the search is methodologically limited by cross sectional or longitudinal designs with follow up of short duration after the surgery [28]. Up until now, a clear correlation has not been identified between craving and weight changes [28]. 

Non-communicable diseases (NCDs) are prevalent in Lebanon and can explain 91% of mortality in the country [29]. Obesity and inactivity are on the rise in the country, with the last World Health Organization (WHO) surveys revealing national prevalence rates of 27% and 36%, respectively [29,30]. Obesity was found to be more prevalent among females and associated with lower socio-economic status [29,31]. BS is on the rise in Lebanon and the Middle East Region, with most centers following the recommendations of international bodies such as the American Society for Metabolic and Bariatric Surgery [32,33]. Yet, changes in diet quality, BED and food craving that occur after such surgeries are poorly studied. In view of this gap, this cross-sectional study, the first of its type in the region, aims to assess the impact of BS on binge eating behaviors, food craving and HEI in Lebanese patients who have undergone such surgery.

## 2. Methods

### 2.1. Patient Population

This cross-sectional study assessed diet quality, binge eating and food craving patterns in adult participants (≥18 years) having undergone BS six months or more prior to the assessment date. Inclusion criteria for the case group (bariatric patients) were as follows: aged 18 years and above, have undergone BS 6 months or more prior to the assessment date and free from any type of confirmed eating disorders. The two most common BS in Lebanon, sleeve gastrectomy and Roux en Y gastric bypass were chosen. To recruit participants, three general surgery physicians performing BS on adult patients and practicing in the areas of Beirut and Mount Lebanon (major referral areas in Lebanon) were contacted to provide a list of patients who had undergone surgery 6 months or more before the time of assessment. Participants were informed of the study goals, as well as the risks and benefits, over the phone. Patients who agreed to be enrolled over the phone were considered to have given consent, and were subsequently assessed in the premises of the Lebanese University in Beirut, Lebanon by two licensed dietitians. Data was collected between September 2017 and April 2018.

Assessment included the collection of information on demographics, surgery type and date, as well as the administration of dietary recalls to calculate HEI scores; the Binge Eating Scale (BES) and the Food Craving Inventory (FCI) were administered as described below. Weight regain was defined as any weight regain after the patient reached their lowest postoperative weight [34,35].

### 2.2. Tools

#### 2.2.1. Healthy Eating Index

On the assessment day, participants were asked to provide a two-day food intake record pre-surgery and another one post-surgery. In these food records, patients were asked to report all the food and beverage items they consumed in a typical weekday and weekend day. The mean of both days represented the participant’s intake preoperatively and postoperatively. These records were transformed into exchanges of food groups and then analyzed to estimate total calories, percentage of saturated fatty acids (SFA), mono-unsaturated fatty acids (MUFA), and poly-unsaturated fatty acids (PUFA), as well as sodium intake and to estimate the score of the HEI components. The HEI 2015 is a measure of diet quality in terms of conformance with the 2015–2020 edition of the Dietary Guidelines for Americans (DGAs) [36]. The 2015–2020 DGAs encourage the consumption of nutrient-dense foods and the reduction of the consumption of refined grains, added sugars, sodium and SFA; higher HEI scores reflect better diet quality. Higher scores are given for increased consumption of total fruits, whole fruits, total vegetables, greens and beans, whole grains, dairy, total-protein food, seafood and plant proteins, fatty acids and for a reduced intake of refined grains, sodium, added sugars and SFA. We followed the simple HEI scoring algorithm method [37]. Each component was analyzed per 1000 calories to provide a relative, rather than absolute, indication of nutrient intake. Fatty acids intake was evaluated using the ratio of unsaturated to saturated fatty acids. The total HEI score was categorized based on numerical scores as follows: 90–100%, Grade A; 80–89%, Grade B; 70–79%, Grade C; 60–69%, Grade D; and 0–59%, Grade F, with grade A reflecting the best diet quality and grade F the worst [36].

#### 2.2.2. Binge Eating Scale

BES is a validated scale to assess BED among bariatric surgery patients [38]. Sixteen questions assess the participant’s emotional, cognitive, and behavioral features [39]. The higher the score, the more severe the disorder, with scores of 0–17 indicating little or no binge eating behavior, scores 18–26 reflecting moderate BED and scores of 27–46 showing severe binge disorder. Among BS patients, a cutoff point of 17 had the highest sensitivity and specificity for predicting BED [38] and was chosen for this analysis.

#### 2.2.3. Food Craving Inventory

The Food Craving Inventory (FCI) is a validated scale to assess the frequency of craving of selected food items. Participants were asked to report on the intake of eight items of each of the following categories: high fat, carbohydrate rich, sweets and fast-food items [40]. Subjects were provided with the definition of food craving and then they were asked about the frequency with which they craved an item in the month prior to the assessment point. Answers could range from one (“never”) to five (“always”).

## 3. Ethical Considerations

This study received approval from the administration of the Lebanese University that had an acting ethics committee role at the time the study was conducted (protocol code CUER # 17 2018, 17 November 2018). The study’s design, conduct and analyses were in line with the Helsinki Declaration of 1975, as revised in 1983.

## 4. Statistical Analysis

Participants’ characteristics were presented as counts (percentages) and means ± SD for categorical and continuous variables, respectively. Variables were characterized by weight regain categories (Yes, No) since they were expected to affect outcomes. Paired differences were assessed using the paired t test for continuous variables. Marginal homogeneity tests and McNemar’s test were used for paired categorical variables that had three or more categories or two categories, respectively. Between group differences were assessed using chi square and Fisher exact test for categorical variables and independent t test for continuous variables. Sensitivity analysis was conducted for the duration of surgery, studying results with or without patients who had undergone surgeries more than five years prior to the assessment date. Logistic regression was performed to assess the factors that were associated with weight regain and BED. Variables that had a *p* value < 0.15 at the univariate level were entered in the multivariate model. Statistical analysis was conducted on IBM SPSS Statistics for Windows (version 25.0, IBM, Armonk, NY, USA).

## 5. Results

A total of 60 subjects participated in the study, most of whom were females (85%) who had undergone sleeve gastrectomy (90%) (Table 1). The sample was relatively young (mean of 35.5 ± 11 years), with a mean duration since BS of 2.4 ± 1.8 years. Most of the participants (95%) had undergone the surgery within the five years preceding assessment. Mean body mass index (BMI) decreased significantly from pre-surgery to the assessment time (43.3 ± 8.0 to 29.3 ± 5.3 Kg/m^2^, *p* < 0.01) and 40% of participants experienced weight regain (Table 1).

Energy intake decreased almost four-fold from a daily intake of 4002 ± 1898 Kcal preoperatively to an intake of 1142 ± 556 Kcal postoperatively. Diet quality improved markedly after the surgery, as seen in the rise in HEI (Table 2).

**Figure 1:**
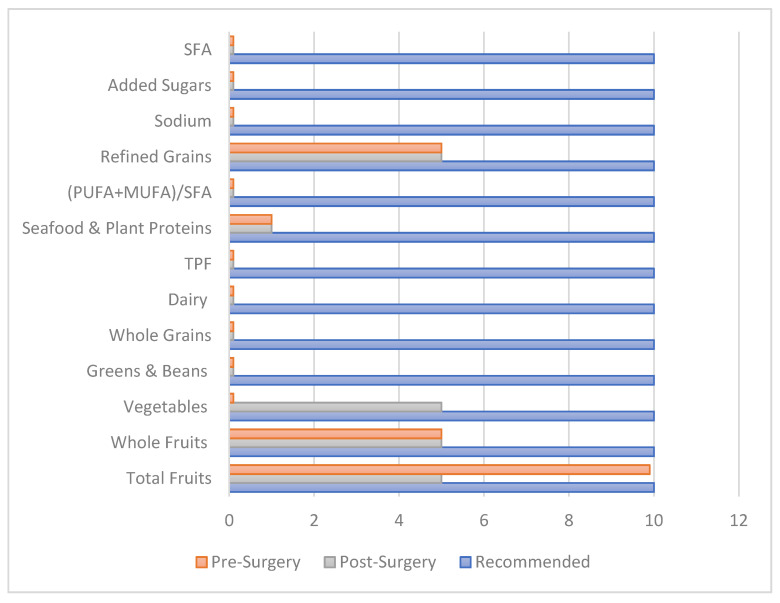
Bar chart of healthy eating index components in the perioperative phase compared to the relevant recommendations. TPF: total protein foods; SFA: saturated fatty acids, PUFA: polyunsaturated fatty acids; MUFA: monounsaturated fatty acids.

Components that contributed to the improvement in diet quality were the enhanced intake of fruits, vegetables, whole grains, dairy and the reduction in the intake of refined grains, sodium and added sugars. Sodium intake increased significantly postoperatively, reaching an intake of 3.1 g/1000 Kcal post operatively (Table 2). Despite the improvement in the HEI continuous score, 97% of the participants remained in the category F, the category with the worst diet quality, compared to only 3% of subjects whose diet quality improved from category F preoperatively to category D postoperatively (data not shown). Most of the components of the HEI did not meet the recommended guidelines after bariatric surgery. Total fruits intake decreased post operatively, yet whole fruits intake did not decrease (Figure 1). This reflects that the reduction was mainly from juice intake. Vegetable intake increased postoperatively, with participants in this category receiving half of the target score (Figure 1).

Components of the HEI were compared across weight regain categories (Table 3). The results revealed that weight regain was not significantly associated with changes in the daily caloric intake and diet quality, as assessed with the HEI components. BES score improved significantly after surgery (Table 4). Similarly, the percent of participants in the severe BES category dropped from 78% to 5% (*p* < 0.01). Food craving followed a similar trend, with scores dropping from 50 ± 36, pre-surgery, to 30 ± 25 post-surgery (*p* < 0.01) (Table 4). Participants had decreased cravings for most confectionaries, baked goods and fast-food items (Appendix A, Table A1). Participants who had a moderate or severe BES had undergone BS earlier, relative to the study period, compared with their healthy peers (3.57 ± 1.9 vs. 2.11 ± 1.6 years, *p* = 0.010). Subgroup analysis by surgery type revealed subjects had similar changes in HEI, BES scores and weight regain. Sensitivity analysis for duration since surgery revealed that results were similar after removing participants who had undergone surgery more than five years prior to the assessment.

Univariate regression revealed that age, educational level, and HEI score post operatively were not significant predictors of weight regain. Multivariate logistic regression, after adjusting for gender, caloric intake postoperatively and time since surgery showed that BES score post operatively was the only significant predictor of weight regain (adjusted odds ratio [AOR]= 1.3 [95% CI:1.1–1.5]). Logistic regression also identified the predictors of a combined moderate and severe BES categories. After adjusting for gender, age and duration since surgery, weight regain was the only significant predictor of BED (AOR = 7.8; 95% CI: 1.3–45.0) (data not shown).

## 6. Discussion

This study assessed diet quality, binge eating and food craving in a sample of BS participants not known to have any eating disorder. Findings revealed that. despite the significant reduction in caloric intake, there was only a shy improvement in HEI score. BES and food craving, nevertheless, improved markedly and weight regain and BED, postoperatively, proved to be highly associated.

Post-surgery, participants had healthier dietary components compared to pre-surgery. They had an increased intake of fruits, whole grains, dairy and a reduction in the energy, refined grains and added sugars’ intakes. Unexpectedly, participants had a reduced intake of vegetables-per-1000-calories, postoperatively. A cross sectional study among BS patients in Lebanon showed participants had an increased intake of vegetables compared to our study findings [41]. These discrepancies in findings may be due to the differences in the dietary assessment tools employed (FFQ vs. 24-h recall), the participants’ baseline characteristics, as well as the follow-up rates.

The HEI allows for an analysis of the conformance of a food group with DGA recommendations. Despite the improvement in continuous HEI scores, 97% patients remained in the HEI category F (worst diet quality), and only 3% of patients had an improvement in HEI categories postoperatively. The slight improvement in the HEI may be due to lack of proper education preoperatively and poor adherence to the advised postoperative education. These results are in line with a recent systematic review on the subject that showed that, despite the reduction in caloric intake, BSs are associated with unbalanced diets, suboptimal protein, vitamin and mineral intakes, and an elevated consumption of fats [42].

Our study revealed that participants had decreased craving for most items including sweet items, with no significant changes in craving scores based on duration since surgery. Reduced cravings for sweet items may be explained by altered taste perceptions postoperatively given that BS may change sweet palatability from favorable to non-favorable and increase sensitivity to sweet taste [43,44]. Interestingly, the literature on the subject revealed that these changes tended to peak in the first 3–12 months postoperatively and to decrease thereafter [45,46]. No changes in craving were noted for items that are rarely consumed in the Middle East region, such as bacon, hot dogs and sausages. Increased sodium intake, postoperatively, was observed among our patient population and has not been well-described in the literature. Other studies have reported a lower sodium intake compared to our patient population [47]. These findings warrant validation and further exploration in future assessments.

Binge eating is the disorder most-affected by BS. This disorder has been described in the literature as “bariatric binge-eating disorder”, as it shares features with the regular disorder as identified with the DSM-V criteria, but with smaller consumed quantities [48]. Binge eating was highly prevalent in our patient population preoperatively, with 95% of subjects classified as moderate or severe bingers. These numbers are much higher than figures from Brazil, which showed a prevalence rate of 42% using the same scale [49] or those found in Italy that revealed a prevalence of 27% using the Symptom Checklist 90-Revised [50]. BES significantly decreased postoperatively, with most patients shifting from the severe to the healthy BES category. The continuous BES score dropped by 21 ± 10 points. This drop is much larger than the one described in a recent prospective cohort study in which the greatest drop in BES observed at one year was a 10-point reduction [51].

Weight regain was identified in 40% of the sample, similar to findings from cohort studies among BS patients [52,53]. Subjects who regained weight had a 60% reduction in energy intake postoperatively. This latter variable predicted weight regain at the univariate scope but lost its significance after accounting for other confounding variables. Similarly, weight regain was not associated with HEI before or after surgery. This contradicts a retrospective study by Da Silva et al., which found that HEI predicted weight regain [11]. These differences may be explained by discrepancies in the definitions used to define weight regain, as well as baseline characteristics such as gender and type of BS [35,54,55]. Even though ‘time since surgery’ has been more evident in the literature as a predictor of weight regain [11,54] this latter variable lost its significance, in our analysis, after adjusting for binge-eating traits. All in all, the only factor that predicted weight regain in our study was BES score. These findings highlight that behavioral evaluation and treatment is a vital component of any obesity-treatment program [56,57]. Psychiatric and behavioral problems have been shown to affect adherence to the advised interventions and to shape BS outcomes [58]. This justifies why BS guidelines promote the integration of mental health professionals in the multidisciplinary care team to support patients in adjusting to the psychological, social and food-behavioral changes experienced in the postoperative phase [59,60]. Finally, in a country such as Lebanon, where inactivity and smoking are major determinants of NCDs, the role of mental health professionals is expected to be the promotion of overall wellbeing by shaping attitudes and behaviors towards exercise, smoking and diet [61].

This study has many limitations. Since our data was collected retrospectively, it is subject to recall bias, as participants were asked to report their diet preoperatively. Moreover, the study lacked an assessment of confounding variables such as physical activity, rates of follow up with the multidisciplinary team, including dietitians, behavioral therapists, and physical trainers. Data on the minimum weight achieved would have given insight on the extent of weight regain. A final limitation relates to the fact that the majority of participants being female, as this may represent a selection bias. Yet, this study has several strengths, despite its small sample size, as it provides insight into the changes in diet quality, binge eating and food craving over a long duration of time. Moreover, participants were interviewed by licensed dietitians who were trained to administer 24-h recall reporting and had insight into diets following BS. Finally, diet recall was changed into indices such as HEI and BES that are used globally, allowing for a wider range of comparison [54,55,57,58].

## 7. Conclusions

This study provided insight into the long-term changes that occur before and after bariatric surgery in a sample of middle eastern Caucasians, presumably free of any eating disorder. The results revealed that, despite the improvement in dietary indicators, HEI improvement remained slight, with most patients categorized in the poor-quality index category. Food-craving and binge-eating scores significantly decreased postoperatively, with more dramatic drops in binge-eating scores as compared with the rest of the literature. Weight regain, prevalent among 40% of the sample, was predicted by binge-eating scores. Our study highlights the importance of intensive preoperative nutritional and psychosocial evaluation for the achievement of short- and long-term care goals. A better understanding of the effect of changes in metabolism perioperatively can also help explain the determinants of weight regain. Future studies should validate the study’s findings in larger samples through prospective dietary as-sessment.

## Figures and Tables

**Table 1:** Characteristics of the study participants.

Variable		Result (*N* = 60)
Gender, *n* (%)	male	9 (15)
	female	51 (85)
Age (years), mean ± SD		35.5 ± 11
Educational Level, *n* (%)	primary	16 (27)
	secondary	12 (20)
	tertiary	32 (53)
Occupation status, *n* (%)	unemployed	24 (40)
	student	10 (17)
	employed	26 (43)
Surgery Type, *n* (%)	bypass	6 (10)
	sleeve	54 (90)
Time since surgery (years)		2.4 ± 1.8
Time since surgery (years)	0.5–1	9 (23)
	1–3	21 (53)
	3–5	8 (20)
	>5	2 (5)
Pre-surgery BMI (Kg/m^2^)		43.3 ± 8.0
Post-surgery BMI (Kg/m^2^)		29.3 ± 5.3
Weight Regain, *n* (%)		24 (40)

BMI: body mass index.

**Table 2:** Healthy index score components prior to and after bariatric surgery (mean ± SD).

Variable	Pre-Surgery	Post-Surgery	*p* Value
Energy Intake (Kcal/day)	4002 ± 1898	1142 ± 556	<0.01
Healthy Eating Index	27 ± 8.6	36 ± 13	<0.01
Total Fruits (cups/1000 Kcal)	0.54 ± 0.83	0.98 ± 1.2	0.015
Whole Fruits (cups/1000 Kcal)	0.53 ± 0.83	0.92 ± 1.2	0.032
Vegetables (cups/1000 Kcal)	0.64 ± 0.57	1.1 ± 1.5	0.025
Greens & Beans (cups/1000 Kcal)	0.06 ± 0.15	0.06 ± 0.17	0.844
Whole Grains (oz/1000 Kcal)	0.05 ± 0.14	0.64 ± 1.0	<0.01
Dairy (cups/1000 Kcal)	0.26 ± 0.25	0.71 ± 1.2	<0.01
TPF (oz/day)	1.6 ± 0.86	2.0 ± 1.4	0.105
Seafood and Plant Proteins (oz/1000 Kcal)	0.88 ± 0.87	1.0 ± 1.7	0.504
$\mathrm{Ratio:}\;\frac{\mathrm{PUFA}\;+\;\mathrm{MUFA}}{\mathrm{SFA}}$	0.67 ± 0.46	0.87 ± 0.83	0.096
Refined Grains (oz/day)	3.3 ± 1.1	2.5 ± 1.4	<0.01
Sodium (grams/1000 Kcal)	2.1 ± 0.48	3.1 ± 2.1	<0.01
Added Sugars (% of energy)	13 ± 8.0	7.1 ± 7.1	<0.01
Saturated Fatty Acids (% of energy)	28 ± 30	24 ± 8.2	0.285

Oz: ounces; TPF: total protein foods; SFA: saturated fatty acids; MUFA: monounsaturated fatty acids; PUFA: polyunsaturated fatty acids.

**Table 3:** Healthy Eating Index changes across weight regain categories (mean ± SD).

Group Comparison	Within Groups						Between Groups	
Variable/Weight Regain Categories	No Weight Regain 36 (60%)			Weight Regain 24 (40%)			*p*-Value Baseline	*p*-Value % Change
	Pre-Surgery	Post-Surgery	% Change	Pre-Surgery	Post-Surgery	% Change		
Daily Energy intake (Kcal)	4010 ± 2100	981 ± 439	71 ± 17	3990 ± 1589	1383 ± 630	60 ± 24	0.968	0.039
HEI score	27 ± 9.0	37 ± 13	−50 ± 65	27 ± 8.3	35 ± 13	−40 ± 68	0.995	0.536
Total Fruits servings/1000 Kcal	0.53 ± 0.88	1.2 ± 1.4	−141 ± 308	0.55 ± 0.78	0.67 ± 0.78	−31 ± 149	0.903	0.213
Whole Fruits (cups/1000 Kcal)	0.52 ± 0.88	1.1 ± 1.4	−145 ± 306	0.54 ± 0.76	0.61 ± 0.78	−17 ± 151	0.912	0.148
Vegetables exchanges, servings/1000 Kcal	0.60 ± 0.56	1.1 ± 1.8	−139 ± 328	0.70 ± 0.58	1.1 ± 1.0	−93 ± 200	0.479	0.560
Greens & Beans (cups/1000 Kcal)	0.08 ± 0.17	0.05 ± 0.19	38 ± 183	0.04 ± 0.13	0.07 ± 0.14	100 ± 0	0.375	0.583
Whole Grains servings/1000 Kcal	0.02 ± 0.09	0.50 ± 0.91	100 ± 0	0.08 ± 0.18	0.85 ± 1.18	−100 ± 246	0.077	0.143
Dairy (cups/1000 Kcal)	0.22 ± 0.22	0.63 ± 0.89	−236 ± 533	0.32 ± 0.29	0.83 ± 1.62	−151 ± 294	0.135	0.514
TPF (oz/day)	1.69 ± 0.83	2.04 ± 1.53	−58 ± 171	1.5 ± 0.92	1.8 ± 1.13	−27 ± 106	0.471	0.445
Seafood & Plant Protein servings (oz/1000 Kcal)	0.93 ± 0.93	0.89 ± 1.33	−61 ± 341	0.79 ± 0.77	1.2 ± 2.1	−34 ± 234	0.547	0.402
$\mathrm{Ratio:}\;\frac{\mathrm{PUFA}\;+\;\mathrm{MUFA}}{\mathrm{SFA}}$	0.64 ± 0.45	1.1 ± 0.99	−150 ± 269	0.72 ± 0.49	0.60 ± 0.41	−32 ± 202	0.550	0.780
Refined Grains (oz/day)	3.1 ± 1.1	2.4 ± 1.4	9.4 ± 68	3.6 ± 1.13	2.8 ± 1.5	8.3 ± 77	0.062	0.058
Sodium (grams/1000 Kcal)	2.2 ± 0.44	3.46 ± 2.41	−67 ± 135	2.1 ± 0.54	2.6 ± 1.5	−34 ± 104	0.536	0.316
Added Sugars (% of energy)	13.4 ± 7.87	6.21 ± 6.38	50 ± 67	12 ± 7. 6	8.5 ± 8.1	6.0 ± 134	0.463	0.103
Saturated Fatty Acids (% of energy)	25 ± 7.7	23 ± 10	−4.7 ± 61	33 ± 46	25 ± 5.8	−11 ± 55	0.281	0.698

HEI: healthy eating index; TPF: total-protein foods. % change = 100* (pre-surgery value − post-surgery value) / pre-surgery value; SFA: saturated fatty acids; MUFA: monounsaturated fatty acids; PUFA: polyunsaturated fatty acids.

**Table 4:** Binge Eating Scale score and Food Craving Index score pre- and post-surgery.

Variable		Pre-Surgery	Post-Surgery	*p* Value
BES score, mean ± SD		31 ± 7.1	10 ± 7.2	<0.01
BES category	None	3 (5)	48 (80)	<0.01
	Moderate	10 (17)	9 (15)	
	Severe	47 (78)	3 (5)	
Food Craving Score, mean ± SD		50 ± 36	30 ± 25	<0.01

BES: binge eating scale.

## Data Availability

All the study data are reported in this paper.

## Appendix A

**Table A1:** Components of the food craving inventory prior and after surgery.

Food Item		Pre-Surgery *N* (%)	Post-Surgery *N* (%)	*p* Value
Cake	0	29 (48)	31 (52)	0.025
	1	6 (10)	12 (20)	
	2	9 (15)	11 (18)	
	3	8 (13)	3 (5)	
	4	8 (13)	3 (5)	
Pizza	0	38 (63)	45 (75)	<0.01
	1	2 (3)	6 (10)	
	2	6 (10)	6 (10)	
	3	3 (5)	3 (5)	
	4	11 (18)	0 (0)	
Fried Chicken	0	38 (63)	45 (76)	<0.01
	1	2 (3)	7 (12)	
	2	6 (10)	3 (5)	
	3	6 (10)	2 (3)	
	4	8 (13)	2 (3)	
Sausages	0	49 (82)	54 (90)	0.020
	1	3 (5)	4 (7)	
	2	2 (3)	1 (2)	
	3	3 (5)	1 (2)	
	4	3 (5)	0 (0)	
Fries	0	37 (62)	47 (78)	<0.01
	1	1 (2)	1 (2)	
	2	3 (5)	4 (7)	
	3	5 (8)	4 (7)	
	4	14 (23)	4 (7)	
Rice	0	43 (72)	46 (77)	0.026
	1	0 (0)	4 (7)	
	2	3 (5)	5 (8)	
	3	7 (12)	3 (5)	
	4	7 (12)	2 (3)	
Hotdogs	0	55 (92)	57 (95)	0.275
	1	1 (2)	1 (2)	
	2	2 (3)	0 (0)	
	3	0 (0)	1 (2)	
	4	2 (3)	1 (2)	
Hazelnuts	0	35 (58)	42 (70)	0.014
	1	3 (5)	3 (5)	
	2	8 (13)	8 (13)	
	3	4 (7)	3 (5)	
	4	10 (17)	4 (7)	
Burgers	0	38 (63)	43 (72)	0.027
	1	2 (3)	4 (7)	
	2	4 (7)	6 (10)	
	3	6 (10)	3 (5)	
	4	10 (17)	4 (7)	
Biscuits	0	42 (70)	45 (75)	0.056
	1	1 (2)	2 (3)	
	2	4 (7)	7 (12)	
	3	3 (5)	4 (7)	
	4	10 (17)	2 (3)	
Ice Cream	0	27 (45)	32 (53)	<0.01
	1	2 (3)	9 (15)	
	2	2 (3)	9 (15)	
	3	11 (18)	3 (5)	
	4	18 (30)	7 (12)	
Pasta	0	38 (63)	41 (68)	0.010
	1	0 (0)	2 (3)	
	2	9 (15)	9 (15)	
	3	4 (7)	3 (5)	
	4	9 (15)	5 (8)	
Fried Fish	0	46 (77)	50 (83)	0.096
	1	2 (3)	0 (0)	
	2	5 (8)	4 (7)	
	3	1 (2)	3 (5)	
	4	6 (10)	3 (5)	
Cookies	0	53 (88)	56 (93)	0.090
	1	2 (3)	2 (3)	
	2	0 (0)	1 (2)	
	3	2 (3)	0 (0)	
	4	3 (5)	1 (2)	
Chocolate	0	19 (32)	21 (35)	0.034
	1	1 (2)	5 (8)	
	2	4 (7)	8 (13)	
	3	2 (3)	5 (8)	
	4	34 (57)	21 (35)	
Pancakes	0	54 (90)	58 (97)	0.144
	1	2 (3)	0 (0)	
	2	2 (3)	0 (0)	
	3	1 (2)	2 (3)	
	4	1 (2)	0 (0)	
Rolls	0	56 (93)	58 (97)	0.050
	2	1 (2)	2 (3)	
	3	2 (3)	0 (0)	
	4	1 (2)	0 (0)	
Donuts	0	47 (78)	49 (83)	0.024
	1	2 (3)	4 (7)	
	2	4 (7)	5 (8)	
	3	4 (7)	1 (2)	
	4	3 (5)	0 (0)	
Candies	0	52 (87)	52 (87)	0.527
	1	1 (2)	1 (2)	
	2	1 (2)	2 (3)	
	3	2 (3)	4 (7)	
	4	4 (7)	1 (2)	
Brownies	0	36 (60)	44 (73)	<0.01
	1	2 (3)	4 (7)	
	2	5 (8)	6 (10)	
	3	6 (10)	2 (3)	
	4	11 (18)	4 (7)	
Bacon *	0	59 (98)	60 (100)	1
	2	1 (2)	0 (0)	
Croissants	0	34 (57)	41 (68)	<0.01
	1	2 (3)	9 (15)	
	2	8 (13)	6 (10)	
	3	9 (15)	3 (5)	
	4	7 (12)	1 (2)	
Steak	0	42 (70)	45 (75)	0.028
	1	2 (3)	5 (8)	
	2	1 (2)	2 (3)	
	3	4 (7)	2 (3)	
	4	11 (18)	6 (10)	
Pie	0	52 (87)	56 (93)	0.029
	1	1 (2)	2 (3)	
	2	5 (8)	0 (0)	
	4	2 (3)	2 (3)	
Baked Potato	0	55 (92)	53 (88)	0.194
	1	1 (2)	0 (0)	
	2	3 (5)	5 (8)	
	3	1 (2)	0 (0)	
	4	0 (0)	2 (3)	
Barbecued Food	0	34 (57)	42 (70)	<0.01
	1	2 (3)	5 (8)	
	2	4 (7)	6 (10)	
	3	6 (10)	2 (3)	
	4	14 (23)	5 (8)	
Mashed Potato	0	58 (97)	55 (92)	0.102
	1	1 (2)	2 (3)	
	2	1 (2)	1 (2)	
	3	0 (0)	1 (2)	
	4	0 (0)	1 (2)	
Bagels	0	54 (90)	57 (95)	0.223
	1	1 (2)	1 (2)	
	2	3 (5)	2 (3)	
	3	0 (0)	0 (0)	
	4	2 (3)	0 (0)	

* Paired assessment of this variable was assessed using McNemar’s test as it had two categories with variables only. The rest of the paired assessments were conducted using Marginal.
